# Speaking the Same Language? A Preliminary Investigation, Comparing the Language and Communication Skills of Females and Males with High-Functioning Autism

**DOI:** 10.1007/s10803-019-03920-6

**Published:** 2019-03-04

**Authors:** Alexandra Sturrock, Natalie Yau, Jenny Freed, Catherine Adams

**Affiliations:** 1 School of Health Sciences, The University of Manchester, Manchester, UK; 2 Department of Human Communication, Development and Hearing, The University of Manchester, Ellen Wilkinson Building, Manchester, M13 9PL UK

**Keywords:** Language and communication, Gender, Autism Spectrum Disorder

## Abstract

Understanding the nature of language and communication disorders in High-Functioning Autism Spectrum Disorder (HFASD) populations may provide insight into why females are more likely than males to go undiagnosed. Language and communication skills were compared between 13 females and 13 males (aged 8.11–11.06) with HFASD. Gender-normative data was also ascertained from 26 typically developing children (TD) matched for age and gender. All children had typical range PIQ, slight variation here was controlled for in analysis. Results show females outperforming males in pragmatic and semantic tasks and in certain language-of-emotion tasks. TDs outperformed HFASDs in above-sentence level tasks, but not in basic vocabulary or sentence level tasks. This study highlights specific strengths/weaknesses in language and communication for female HFASD, which could aid more accurate identification of the female autistic phenotype. It indicates the need for larger follow up studies in this area.

**Electronic supplementary material:**

The online version of this article (10.1007/s10803-019-03920-6) contains supplementary material, which is available to authorized users.

## Introduction and Background

A substantive proportion of children and young people with High Functioning Autism Spectrum Disorder (HFASD) have language and communication problems (Kissine et al. 2016). Skills in these areas are crucial in gaining and sustaining peer relationships, education and employment success (Howlin et al. 2004). Language deficits in HFASD are also associated with developing anxiety and other mental health conditions (Mayes and Calhoun 2011). The timely identification and diagnosis of children with ASD entails optimal access to information and practical support for developmental needs (Calzada et al. 2012). Females who meet criteria for ASD are more likely than males to go undiagnosed, be mis-diagnosed with another disorder or be referred to autism services later in life (Dworzynski et al. 2012; Giarelli et al. 2010). Rates of diagnosis are especially poor for females with typical range (70+) Intelligence Quotient (IQ) (Nicholas et al. 2008), despite elevated social and emotional vulnerabilities (Bargiela et al. 2016). It has been suggested that differences in presentation of ASD, including structural language and pragmatic abilities, may obscure underlying difficulties and mean females are less likely to be referred to diagnostic services (Kenyon 2014). Therefore, a more thorough understanding of the differences in male/female language and pragmatic profiles in ASD is critical to understand the specific needs of both groups, and whether effective intervention is being denied to an undiagnosed female population.

Currently, diagnosis of autism relies on clinical observations and reports of behaviour, which represents underlying difficulties in core domains of social interaction, communication, and restricted, stereotyped, repetitive behaviours (RSR) (WHO 1994). However, recent studies have shown females with ASD to have a different profile of social skills (Head et al. 2014) and RSR behaviours (Szatmari et al. 2012; Mandy et al. 2012). This could indicate a distinct female phenotype in autism (Van Wijngaarden-Cremers et al. 2014). In the current study, we aim to add to the understanding of the female with ASD phenotype, by using in-depth linguistic and pragmatic tasks to explore gender differences. We focus on the most diagnostically under-represented sub-group, females with HFASD, and make comparisons to males with HFASD and gender-matched controls with typical development (TD) (females: FwTD/males: MwTD).

Until recently research into the presentation of ASD between genders has focused on social interaction and RSR behaviours, with communication relatively under-investigated. Females with HFASD are rated better overall than males with HFASD in the domain of social interaction, using diagnostic checklists (Hartley and Sikora 2009). Females with ASD are also likely to report being more socially driven, tend to have closer and more reciprocal friendships than male peers (Sedgewick et al. 2016), and better pretend play skills (Zwaigenbaum et al. 2012) than males with HFASD. However, these relatively spared skills for females with HFASD may lead to underestimates of the functional difficulties this group experience. When compared to TD gender-matched peers, females with HFASD have demonstrated lower levels of emotional reciprocity (Head et al. 2014), and reduced capacity to identify emotions from pictures (Lai et al. 2012). It is therefore possible that females with HFASD experience their difficulties equally severely as males with HFASD, when compared to their typically-developing female friendship groups. These relative difficulties may explain increased reporting of perceived problems by females with HFASD and their families (Holtmann et al. 2007; Lai et al. 2011). It is important, therefore, in any assessment of language and pragmatic skills of females with HFASD, to make comparisons not only to males with HFASD (where they are likely to show strengths) but also to females with TD, where functional deficits in gender peer groups can be evidenced (Lai et al. 2015).

Previous comparative studies of HFASD and TD language have found similar levels of performance on tasks of expressive and receptive vocabulary (Howlin 2003; Kjelgaard and Tager-Flusberg 2001; Kelley et al. 2006). However, sentence level skills (e.g., sentence comprehension) were more likely to be impaired than vocabulary in HFASD (Kjelgaard and Tager-Flusberg 2001). Language at above-sentence level (e.g., narration, discourse, comprehension) is relatively poorly researched in HFASD, despite common reporting of functional difficulties in this area (Attwood 2007). Pragmatics is relatively well researched in HFASD, but studies tend to focus on observation of pragmatic behaviour in interaction (e.g., Geurts and Embrechts 2008), and less frequently focus on the ability to process pragmatic information (e.g. inference).

In typically developing populations, there tends to be a subtle advantage to females over males in terms of language and social communication. In early years TD males are more likely to have delayed first words, have a smaller vocabulary and less grammatically complex sentences (Eriksson et al. 2012; Bouchard et al. 2009). This finding has more recently been replicated in an ASD population (Kozlowski and Matson 2012). In male TD populations, structural language skills (vocabulary and sentence grammar) are thought to catch up with females by mid-childhood, although females are likely to demonstrate better pragmatic skills (i.e. social use of language) (Leaper 1991; Ladegaard and Bleses 2003) and use longer and more complex grammatical structures in spontaneous communication throughout teenage and adult life (Mulac 2009). It is probable that this distinction is replicated in the ASD group, although there is little evidence to date. Where gender comparisons of language in ASD have been conducted, methodological differences in study design have impacted heavily on the interpretation of results. Research selecting female participants from a diagnosed population tends to over-represent those with lower IQ (Lai et al. 2015). Language studies where IQ has not been controlled for has resulted in an over-representation of language problems being identified for the females with ASD group (Lord et al. 1982; Tsai and Beisler 1983). When this group is controlled for IQ, females with HFASD have typically been found to have communication skills similar to male peers (Mandy et al. 2012; Solomon et al. 2012) or better (Park et al. 2012; Lai et al. 2011).

Investigations using measures of parental report and diagnostic tools have generally not found evidence of gender differences in HFASD language development (Dworzynski et al. 2012; Mandy et al. 2012; Solomon et al. 2012). However, these items show methodological limitations. For example, diagnostic tools may be poor at representing females with HFASD language difficulties, as they are thought to over-represent the male phenotype (Kreiser and White 2014). In addition, data collected from parental report may find parents of females with HFASD over-reporting language and communication difficulties when compared to male peers, limiting measures of difference between genders (Holtmann et al. 2007). Wide ranging participant ages evident in many studies may reduce effective comparison of between group or gender difference due to the likely correlated range of developmental language levels (Hull et al. 2017). Despite limiting factors of existing studies, some differences in social communication skills have been identified, with females with HFASD showing fewer difficulties in this domain than males with HFASD (Park et al. 2012; Lai et al. 2011; Zwaigenbaum et al. 2012). Parental reports have also documented qualitative differences in social communication, with females prone to excessive talking, echolalia in childhood and questioning in older years, and greater likelihood to adopt different voices when talking to others (Kopp and Gillberg 1992, 2011).Further, Goddard et al. (2014) demonstrated that female HFASD outperformed male HFASD on an isolated language measure of category naming. There is currently no research evidencing gender differences in HFASD on pragmatic measures.

A deficit in understanding and expressing the language of emotion is a primary characteristic of HFASD (Frith 1991). Various studies identify difficulties with mental state vocabulary (Kelley et al. 2006; Ziatas et al. 1998) and generating emotional vocabulary items in spontaneous storytelling (Perlman-Avnion and Eviatar 2002; Sillar et al. 2014). Others point to difficulties in identifying emotions (Hobson and Lee 1989) and naming emotions from pictures (Lindner and Rosen 2006). There is currently no data on differences between females and males with HFASD on measures of emotion language. In typical developing children, females are more likely than males to use basic mental state verbs (e.g., *to like*) by 30 months (Bouchard et al. 2009) and later will use language more frequently to express their own internal states (thoughts, emotions and senses; Thompson and Moore 2000; Newman et al. 2008). If these differences are mirrored in the HFASD population, females with HFASD should perform better than males with HFASD in receptive and expressive tasks associated with emotion vocabulary.

### Current Study

In the current study, we adopt an in-depth approach to the assessment of communication skills by diagnosis and gender, based on contemporary experimental methods in the study of language acquisition and in clinical testing of language structure and function. In addition, we control our participants for age and performance IQ, to allow detailed comparison of key variables across genders (females vs. males) and diagnostic (HFASD vs. TD) groups. We aim to use results to provide detail about what might constitute a female with HFASD profile of language and communication skills. The language measures used and specific hypotheses are as follows: In general we anticipate that TD groups will outperform HFASD groups on most language tasks. We make the following predictions regarding gender differences in four main areas of language and communication: (1) basic structural language (receptive and expressive measures at word and sentence level): we predict minimal gender differences on measures of basic structural language in either the HFASD or TD group pairs; (2) above-sentence level structural language: it is hypothesised that there will be better performance by females with HFASD over males with HFASD on above-sentence level language tasks; (3) measures of pragmatics and semantics (knowledge of word meanings) and (4) measures of emotion vocabulary (understanding, spontaneous use in narrative and emotion word generation): we predict gender differences on measures (3) and (4), with females with HFASD outperforming males with HFASD in all areas.

## Methods

### Participants

Thirteen female and thirteen male children with HFASD were recruited through participating UK National Health Trusts, local autism charities and private educators. Inclusion criteria were; performance IQ over 70, age between 9y0m and 10y11m and evidence of multi-disciplinary ASD diagnostic assessment using DSM (APA 2013) or ICD (WHO 1994) criteria and scores above cut-off on the Autism Spectrum Screening Questionnaire (ASSQ) (Ehlers et al. 1999). ATD group (*n* = 26) with normal range IQ was matched on age and gender to the HFASD children. TD participants were recruited through the UK ESRC International Centre for Language and Communication Development (LuCiD) research group and database. These children fell below published cut-off scores using the ASSQ (Ehlers et al. 1999). The final age range for all participants was extended to 8y11m to 11y6m to improve recruitment, which was especially difficult for the females with HFASD group. PIQ (range 75–139, M: 111.85) and age in months (range 107–138, M:123.37) were compared between gender and diagnostic groups: PIQ but not age showed a small but significant difference and was therefore controlled for within analysis. All children had English as a first language and no uncorrected hearing or visual impairment. In order to maximise recruitment, individuals with confounding co-morbidities were not excluded from the participant numbers. Co-occurring difficulties were only identified in the HFASD group, and included ADHD and anxiety; both were managed through taking breaks, splitting sessions and offering reassurance. In two cases children with HFASD who had originally agreed to participate in the study elected to discontinue due to anxiety; test numbers do not include their data. Two children with autism failed to comply with test requirements on one measure, reducing the HFASD participant numbers from 26 to 25 on two measures. Screening assessments were administered by trained clinicians/researchers during an initial visit. Both were highly experienced in presenting psychological and/or language assessments to individuals with HFASD.

### Procedure

Children were seen individually at their home or school. Typically, language assessments were completed in two sessions of 60 min each. Some flexibility was allowed for children with lower levels of attention and anxiety; for example, providing play breaks and/or shorter but more numerous sessions (maximum: 6 sessions × 30 min). Table 1 shows the fixed order of tasks presented. The procedures for established assessments were derived directly from published guidelines. The procedure for novel and experimental measures are detailed below, and task development is outlined in the appendices. Narration and samples of discourse were video recorded; sentence recall and semantic word association tasks were audio recorded for subsequent analysis.

**Table 1:** Order of test measures and domain of language assessment

Test order	Assessment name	Domain assessed
Session 1		
1	Wechsler Abbreviated Scale of Intelligence (WASI; Weschler 1999)	Performance IQ inclusion criteria
2	The Autism Spectrum Screening Questionnaire (ASSQ: Ehlers et al. 1999)	Autism inclusion criteria
Session 2		
3	British Picture Vocabulary Scale (BPVS-3) (Dunn et al. 1997)	Basic structural language: receptive vocabulary
4	Receptive emotion vocabulary (REV): novel task	Language of emotion: Matching emotion words to picture
5	The Clinical Evaluation of Language Fundamentals—fourth edition (CELF-4): recalling sentences subtest (Semel et al. 2006)	Measure of basic structural language: expressive grammatical knowledge
6	CELF-4 Word Associations subtest	Semantics: word generation with category
7	Emotions word association task	Language of emotion: word generation with emotion category
Break		
8	Sensitivity to Grammatical Errors task (Eigsti and Bennetto 2009)	Basic structural language: receptive grammatical knowledge
9	CELF-4 Understanding Spoken Paragraphs subtest	Above-sentence level structural language: discourse comprehension
Session 3		
10	The Test of Receptive Grammar—second edition (TROG-2) (Bishop, 2003)	Basic structural language: receptive grammar
11	Figurative Language task (MacKay and Shaw 2004)	Pragmatics: receptive pragmatic task
12	Local Coherence Inference task (Joliffe and Baron-Cohen 1999)	Pragmatics: receptive pragmatic task
Break		
13	The Test of Word Knowledge (TOWK) expressive vocabulary subtest (Wiig and Secord 1992)	Basic structural language: expressive vocabulary
14	Expressive narrative task	Above-sentence level structural language: expressive above sentence level grammar Pragmatics: Coherence in Narrative Language of emotion: spontaneous generation of vocabulary

The order of assessment was not altered for children with lower attention levels. Session 2 prior to the break was always kept intact due to the progression of test items

### Control Measures

#### *Wechsler abbreviated scale of intelligence (WASI; Weschler*^1999^*)*

Performance IQ obtained by using two subtests for block design and matrix reasoning.

#### *The Autism Spectrum Screening Questionnaire (ASSQ: Ehlers et al. *1999*)*

A 27 item screening tool designed to identify diagnostic features of autism, with particular validity for participants with typical range IQ.

### Measure of Basic Structural Language

The following measures were chosen for their frequent use in clinical settings and validity in identifying difficulties at single word and sentence level. Permitted age ranges for test use are shown for each measure. *British Picture Vocabulary Scale (BPVS-3)* (Dunn et al. 1997): the child demonstrates receptive word knowledge by identifying a target word from a choice of four pictures following a spoken presentation of the word target by the assessor (3–16 years).*The Test of Word Knowledge (TOWK) Expressive Vocabulary subtest* (Wiig and Secord 1992): the child demonstrates expressive vocabulary by generating and producing a word in response to the assessor asking a question, such as ‘what is this?’ and pointing at a picture (5–17 years). *The Test of Receptive Grammar—second edition (TROG-2)* (Bishop 2003): The assessor presents a sentence and the child finds the picture which best fits from a choice of four. More complex grammatical forms include comprehension of subordinate clause forms, e.g., find ‘the scarf the book is under is blue’ (4 years to adult). *The Clinical Evaluation of Language Fundamentals—fourth edition (CELF-4) Recalling Sentences subtest* (Semel et al. 2006): The child is presented with a spoken sentence and is asked to recite this verbatim. Errors made by the child were tallied to produce a raw and standardised score. Subtest items gradually get more complex, e.g., from: “the tractor was followed by the bus”, to: “Before the students were dismissed for lunch, they were told by the teacher to turn in their assignments”. *Sensitivity to Grammatical Errors task (SGE)* (Eigsti and Bennetto 2009): The child demonstrates awareness of grammatical errors at sentence level by identifying whether a sentence is correct or incorrect following a spoken stimulus. There are 38 items which comprise 19 pairs of grammatically correct or incorrect sentences (example in Online Appendix 1). This experimental measure was chosen because it demonstrated difference between groups of children with HFASD and TD. It was adapted to a shorter subset of test items in order to manage demand on the children across the assessment session (details in Online Appendix 1).

### Measures of Above-Sentence Level Structural Language

The following measures were chosen to identify above-sentence level structural language differences between HFASD and TD controls: *The Clinical Evaluation of Language Fundamentals—Fourth Edition (CELF-4): Understanding Spoken Paragraphs subtest* (Semel et al. 2006): The child listens to three stories presented by the assessor and then demonstrates comprehension of above-sentence level text by answering five questions which entail comprehension of the whole text. Answers correct were tallied to produce a raw and standardised score. *Expressive Narrative*: The child was asked to familiarise themselves with the picture book, “A boy, his dog and a frog” (Mayer 2003). They were then asked to tell the story, using the book as reference. The narrative was video recorded, transcribed and analysed for story length (in words) and language complexity (a composite score of temporal markers and causal markers). Number and range of markers were also recorded; e.g., if the temporal marker ‘and then’ is repeated 21 times this yields a score 21 for number and 1 for range. Overall scoring criteria were established with reference to Petersen et al. (2008) and Kelley et al. (2006). The same narrative sample was analysed for the Coherence in Narrative task and spontaneously generated language of emotion tallies (see below).

### Measures of Pragmatic and Semantic Ability

#### *Pragmatics: Figurative Language Task (MacKay and Shaw *2004*)*

**Fig. 1:**
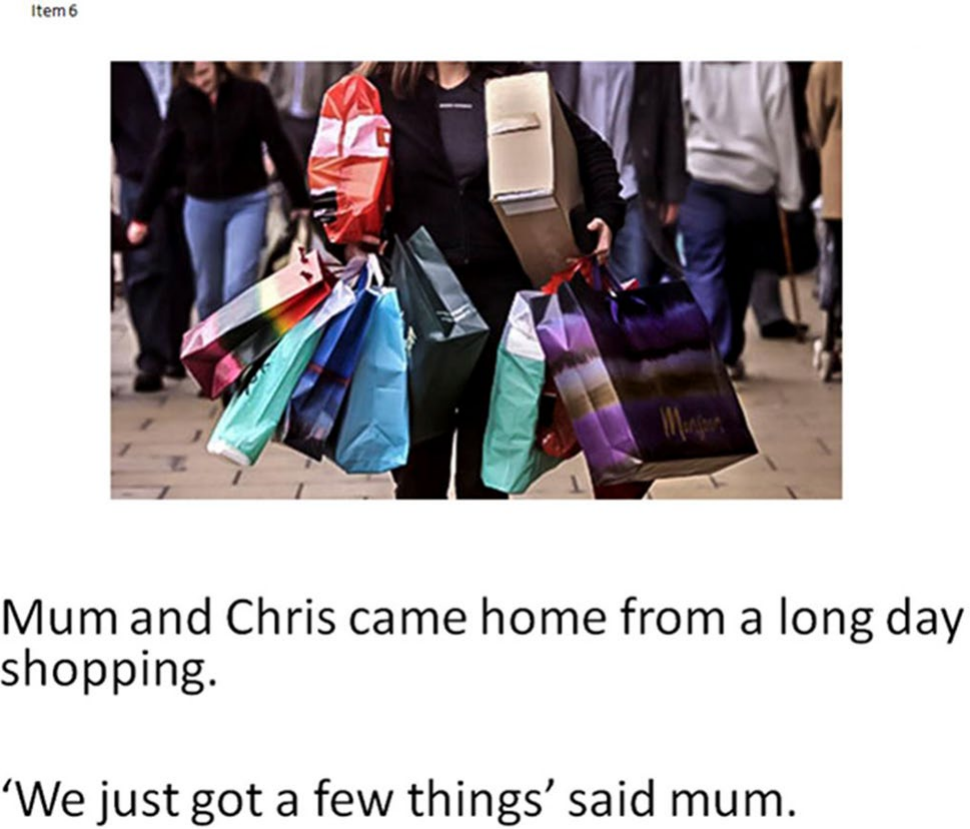
Example of figurative language task based on MacKay and Shaw (2004)

An experimental measure of 21 items testing understanding of figurative language (meaning and intention behind usage). This measure was chosen because of its previous success evidencing difference between children with HFASD and TD. Test items cover three examples of each; irony, hyperbole, metonym, indirect comment, rhetorical question, understatement and metaphor. Metaphor was added in our adapted version in order to provide a thorough coverage of figurative language types. Other adaptations in presentation were made to manage demands for the children over the assessment sessions (details in Online Appendix 2). The participants are presented with an example of figurative language and a picture which provides contextual information to support accurate interpretation (Fig. 1). The child is asked to describe the true meaning of the figurative language and what intention the speaker had for using a non-literal phrase. One point was given for each correct response in line with original study scoring criteria.

#### *Pragmatics: Local Coherence Inference Task (Joliffe and Baron-Cohen *1999*)*

**Fig. 2:**
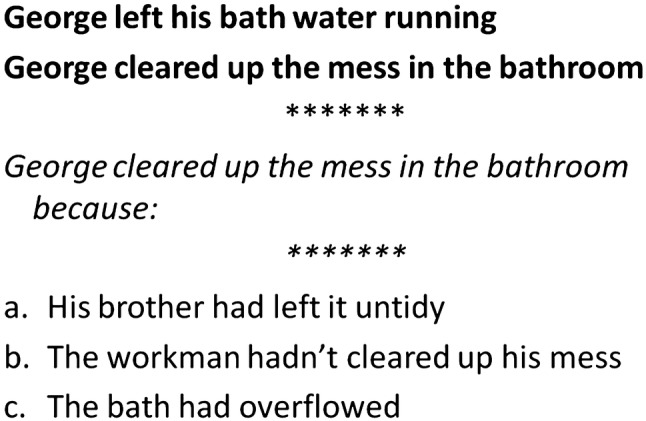
Example of local coherence inference task based on Joliffe and Baron-Cohen (1999)

An 18 item experimental measure, testing understanding of inferred meaning which provides coherence to a short story. This test measure was selected due to previous evidence of demonstrating difference between adults with HFASD and TD. Original material was modified to suit the younger participants in our study (for details see Online Appendix 3). The child reads a short story which purposely omits an overt bridging reference between an initiating event and a consequence (Fig. 2). The child is asked to correctly identify the missing information from a choice of three, all of which could be appropriate but one constitutes the best fit. Responses were scored correct/incorrect and timed between the end of reading the story and selection of an option.

#### *Pragmatics: Coherence in Narrative*

An elicited narrative sample scoring for the child’s expressive use of coherent features in storytelling. Transcriptions taken during the *Expressive Narrative task* (see above for “Methods”) were scored for story complexity (criteria by Demir et al. 2015). This was rated on a 6 point rising scale (1) descriptive narrative only, (2) includes an action sequence, (3) showing a reactive sequence but with no goal, (4) has a goal but no conclusion to action, (5) covers one complete episode of goal-driven action and conclusion, (6) includes multiple episodes of goal-driven action and integrated conclusion. Transcription and reliability were validated by a second-rater; disagreements in results were discussed until agreement was reached.

#### *Semantics: The Clinical Evaluation of Language Fundamentals—Fourth Edition (CELF-4): Word Associations subtest (Semel et al. *2006*)*

The child is asked to generate words within super-ordinate categories of animals, food, and occupations, following the instruction: “Name different jobs or occupations that people might have. Name as many as you can in 1 min. For example, you could say *babysitter* or *mechanic*. Now you name some more. Start now” Raw scores were generated for each category.

### Measures of Language of Emotion

#### *Receptive Emotion Vocabulary (REV)*

**Fig. 3:**
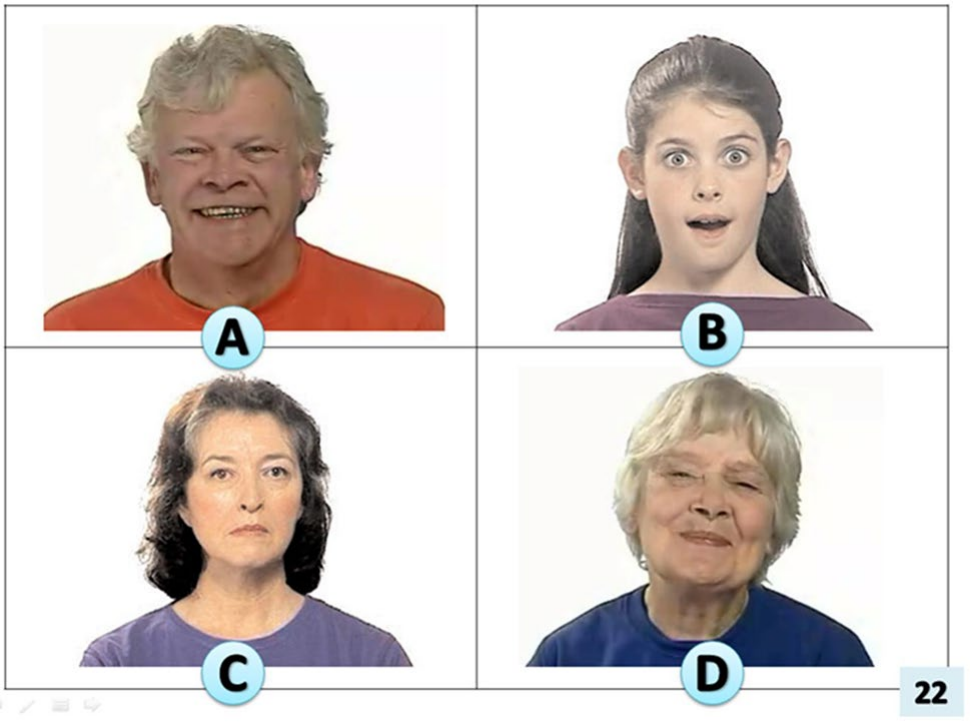
Example of visual stimuli for novel receptive emotion vocabulary task

A novel measure in which the child identifies a target emotion vocabulary item from a choice of four pictures (see Fig. 3), following a spoken presentation of the word target by the assessor. Thirty-nine test items were developed to cover the receptive vocabulary of emotion over a range of ages and language abilities. This measure was developed to provide a focused assessment covering the receptive vocabulary of emotion over a range of ages and language abilities. This language parameter had not been covered by existing measures. Details of test development are in Online Appendix 4. Responses were scored one for each test item correct and it was noted whether incorrect choices fell into the *close* or *distant* distracter categories, therefore showing detail in the variation of difficulty.

#### *Spontaneous Emotion Vocabulary in Narrative (Number and Range)*

Using transcriptions of narrative from the *Expressive Narrative task* (see above), examples of emotion vocabulary were tallied for total number and range.

#### *Word Association Task for Language of Emotions*

A novel task which follows the protocol used in the CELF-4: Word Association subtest, but using emotion as the super-ordinate category. It was developed to measure a parameter of emotion vocabulary which was not addressed in existing test measures. The instruction is to “Name different feelings or emotions that people might have. Name as many as you can in 1 min. For example, you could say *happy* or *embarrassed*. Now you name some more. Start now”. The task generates raw scores, one for each correct item. Details of task pilot are in Online Appendix 5.

## Results

Twenty-six children with HFASD (female n = 13; male n = 13) and twenty-six children with TD (female n = 13; male n = 13) took part in all assessments. Missing data occurred for one MwHFASD^1^ on the test of Sensitivity to Grammatical Errors, one FwHFASD on the Test of Word Knowledge (expressive vocabulary); one FwTD and one MwTD narrative recordings quality limited scoring. Group differences on control measures (chronological age and performance IQ: PIQ), identified in Table 2, were analysed first using a 2 (Gender) × 2 (Group) analysis of variance (ANOVA). There was a small but significant main effect of Group on PIQ (*F*(1,48) = 0.072, *p* = .021, ŋ^2^ = 0.105) as the TD group scored significantly higher (mean = 116.62) than the HFASD group(mean = 107.08).There were no other significant effects on the PIQ measure (Gender: *F*(1,48) = 0.072, *p* = .790, ŋ^2^ = 0.001; Group × Gender interaction: *F*(1,48) = 0.001, *p* = .970, ŋ^2^ = 0.000). The groups were well-matched for chronological age: Group (*F*(1,48) = 2.924, *p* = .094, ŋ^2^ = 0.057); Gender (*F*(1, 48) = 1.634, *p* = .207, ŋ^2^ = 0.033); Group × Gender interaction (*F*(1,48) = 1.898, *p* = .175, ŋ^2^ = 0.038).

**Table 2:** Descriptive statistics for chronological age (in months) and PIQ scores for HFASD and TD groups by gender

	HFASD (*n* = 26) Mean (SD)	TD (*n* = 26) Mean (SD)	Gender overall Mean (SD)
PIQ (raw score)			
Female	107.69 (17.32)	117.08 (14.95)	112.38 (16.56)
Male	106.46 (11.93)	116.15 (13.10)	111.31 (13.23)
Group overall	107.08 (14.59)	116.62 (13.78)	111.85 (14.85)
Age (in months)			
Female	124.46 (8.35)	125.23 (6.98)	124.85 (7.55)
Male	118.31 (9.93)	125.46 (7.88)	121.88 (9.51)
Group overall	121.39 (9.52)	125.35 (7.29)	123.37 (8.63)

Group differences on all study measures were subsequently examined using a series of 2 (Gender) × 2 (Group) ANOVAs. These are reported below, beginning with the basic structural language assessments and following the same order as the list of assessments in “Methods”. Raw scores were used for all analyses. Standard scores are reported in tables where appropriate, to illustrate how scores relate to typical performance on standardised measures. Given that HFASD and TD groups differed significantly on the PIQ measure, any significant group differences (*p* < .05) were followed up by ANCOVA to explore whether those differences could be explained by differences in PIQ.

### Measures of Basic Structural Language

Descriptive statistics (means and standard deviations) for basic structural language tests and experimental tasks are shown in Table 3. No analysis was pursued with data from the Test of Receptive Grammar (TROG-2), since there were ceiling effects on this measure.

**Table 3:** Descriptive statistics on basic structural language assessments for HFASD and TD groups by gender

	HFASD (n = 26) Mean (SD)		TD (n = 26) Mean (SD)		Gender overall Mean (SD)
	Raw score	Standard score	Raw score	Standard score	
BPVS-3: Receptive vocabulary (max. score 132)					
Female	106.69 (16.34)	104.38 (16.95)	111.31 (5.63)	111.00 (6.53)	109.00 (12.20)
Male	104.69 (16.65)	107.15 (14.63)	116.08 (7.70)	115.46 (5.94)	110.38 (13.97)
Group overall	105.69 (16.19)		113.69 (7.04)		109.69 (13.01)
TOWK: Expressive vocabulary (max. score 29)	(n:25)				
Female	22.54 (3.46)	13.00 (3.42)	23.08 (2.84)	13.25 (2.53)	22.80 (3.12)
Male	20.92 (4.65)	12.69 (3.95)	23.69 (3.33)	13.92 (2.78)	22.31 (4.20)
Group overall	21.73 (4.09)		23.40 (3.06)		22.55 (3.68)
TROG-2: Receptive grammar (max. score 80)					
Female	75.46 (5.47)	112.69 (6.65)	76.31 (2.59)	112.92 (6.40)	75.88 (4.22)
Male	72.85 (7.18)	111.92 (12.83)	77.00 (3.67)	115.00 (4.87)	74.92 (5.97)
Group overall	74.15 (6.39)		76.65 (3.14)		75.40 (5.14)
Sensitivity to grammatical errors task (max score 38)	(n:25)				
Female	35.69 (2.25)		34.00 (3.33)		34.88 (2.89)
Male	35.85 (1.35)		35.92 (1.26)		35.88 (1.28)
Group overall	35.77 (1.82)		35.00 (2.61)		35.39 (2.26)
CELF-4 Recalling Sentences subtest (max score 95)					
Female	68.85 (10.13)	10.69 (2.81)	72.23 (12.71)	11.46 (3.20)	70.54 (11.39)
Male	60.69 (13.76)	9.08 (3.01)	74.23 (10.83)	11.77 (2.35)	67.46 (13.96)
Group overall	64.77 (12.55)		73.23 (11.61)		69.00 (12.71)

Receptive Vocabulary (BPVS-3): There was a small but significant main effect of Group (*F*(1, 48) = 5.241, *p* = .026, ŋ^2^ = 0.098) as the TD participants (mean = 113.69) outperformed the HFASD participants (mean = 105.69). This was no longer significant when controlling for PIQ (*F*(1, 47) = 1.892, *p* = .176, ŋ^2^ = 0.039). There was no significant effect of Gender (*F*(1, 48) = 0.157, *p* = .694, ŋ^2^ = 0.003) and no significant Group x Gender interaction (*F*(1, 48) = 0.938, *p* = .338, ŋ^2^ = 0.019). There were no significant effects on the Expressive vocabulary subtest (TOWK) (Group: *F*(1, 47) = 2.634, *p* = .111, ŋ^2^ = 0.053; Gender: *F*(1, 47) = 0.243, *p* = .624, ŋ^2^ = 0.005; Group × Gender: *F*(1, 47) = 1.187, *p* = .282, ŋ^2^ = 0.025) or on the Sensitivity to Grammatical Errors task ((Group: *F*(1, 47) = 2.889, *p* = .096, ŋ^2^ = 0.058; Gender: *F*(1, 47) = 1.748, *p* = .193, ŋ^2^ = 0.036; Group × Gender: *F*(1, 47) = 2.097, *p* = .154, ŋ^2^ = 0.043)).

Recalling Sentences subtest (CELF-4/RS): There was a small and significant main effect of Group (*F*(1, 48) = 6.525, *p* = .014, ŋ^2^ = 0.120) as the TD participants answered more questions correctly (mean = 73.23) than the HFASD participants (mean = 64.77). This was no longer significant after controlling for PIQ (*F*(1, 47) = 2.893, *p* = .096, ŋ^2^ = 0.058). Neither the main effect of Gender (*F*(1, 48) = 0.863, *p* = .358, ŋ^2^ = 0.018) or the Group × Gender interaction was significant (*F*(1, 48) = 2.349, *p* = .132, ŋ^2^ = 0.047).

Overall, TD children showed better receptive vocabulary and better ability to recall sentences than children with HFASD. However, this effect disappeared after controlling for PIQ and there were no significant differences between genders. There was no evidence that FwHFASD show a different profile of basic structural language ability than MwHFASD using these measures.

### Measures of Above-Sentence Level Structural Language

Descriptive statistics (means and standard deviations) for CELF-4 Understanding Spoken Paragraphs subtest and Expressive Narrative markers (story length, language complexity, temporal markers number and range, causal markers number and range) are reported in Table 4.

**Table 4:** Descriptive statistics on higher level structural language tasks for HFASD and TD groups by gender

	HFASD (n = 26) Mean (SD)	TD (n = 25) Mean (SD)	Gender overall Mean (SD)	Significance on measure		
	Raw score	Raw score		Group	Gender	Interaction
CELF-4 Understanding spoken paragraphs subtest (max score 15)		(n:26)		*p* = .303	*p* = .146	*p* = .214
Female	12.15 (2.76)	12.00 (2.24)	12.08 (2.47)			
Male	10.23 (2.77)	11.85 (2.30)	11.04 (2.63)			
Group overall	11.19 (2.89)	11.92 (2.23)	11.56 (2.58)			
Expressive narrative—story length (max score 527)				*p* = .081	*p* = .140	*p* = .569
Female	253.23 (101.03)	323.83 (93.12)	287.12 (101.85)			
Male	225.46 (134.31)	261.75 (84.81)	242.88 (112.51)			
Group overall	239.35 (117.29)	292.79 (92.70)	265.00 (108.537)			
Expressive narrative—language complexity (max score 35)				*p* = .003	*p* = .268	*p* = .796
Female	10.23 (7.38)	16.58 (8.36)	13.28 (8.35)			
Male	7.15 (6.68)	14.67 (8.97)	10.76 (8.60)			
Group overall	8.69 (7.08)	15.63 (8.54)	12.02 (8.49)			
Expressive narrative—temporal markers number (max score 26)				*p* = .059	*p* = .525	*p* = .897
Female	8.92 (5.94)	12.17 (6.52)	10.48 (6.33)			
Male	7.54 (6.50)	11.25 (6.40)	9.32 (6.59)			
Group overall	8.23 (6.14)	11.71 (6.36)	9.90 (6.42)			
Expressive narrative—temporal markers range (max score 6)				*p* = .026	*p* = .470	*p* = .187
Female	2.69 (1.49)	3.08 (1.31)	2.88 (1.39)			
Male	1.85 (1.86)	3.33 (0.88)	2.56 (1.63)			
Group overall	2.27 (1.71)	3.21 (1.10)	2.72 (1.51)			
Expressive narrative—causal markers number (max score 22)				*p* = .014	*p* = .036	*p* = .942
Female	1.77 (1.69)	4.42 (3.58)	3.04 (3.02)			
Male	1.00 (1.00)	3.50 (6.02)	2.20 (4.33)			
Group overall	1.38 (1.42)	3.96 (4.87)	2.62 (3.72)			
Expressive narrative—causal markers range (max score 6)				*p* = .003	*p* = .006	*p* = .089
Female	0.92 (0.49)	1.83 (0.94)	1.36 (0.86)			
Male	0.69 (0.63)	1.25 (1.14)	0.96 (0.94)			
Group overall	0.81 (0.57)	1.54 (1.06)	1.16 (0.91)			

There were no significant group effects (diagnosis and gender) for CELF-4 Understanding Spoken Paragraphs subtest (Group: *F*(1, 48) = 1.083, *p* = .303, ŋ^2^ = 0.022); Gender: (*F*(1, 48) = 2.187, *p* = .146, ŋ^2^ = 0.044); Group × Gender: *F*(1, 48) = 1.587, *p* = .214, ŋ^2^ = 0.032 or for Narrative Temporal Markers number: Group: *F*(1, 46) = 3.745, *p* = .059, ŋ^2^ = 0.075; Gender: *F*(1, 46) = 0.410, *p* = .525, ŋ^2^ = 0.009; Group × Gender: *F*(1, 46) = 0.017, *p* = .897, ŋ^2^ = 0.000.

On other Expressive Narrative separate indices, findings were more mixed. There were no significant effects for Narrative Story Length: (Group: *F*(1, 46) = 3.194, *p* = .081, ŋ^2^ = 0.065); Gender: *F*(1, 46) = 2.257, *p* = .140, ŋ^2^ = 0.047; Group × Gender: *F*(1, 46) = 0.329, *p* = .569, ŋ^2^ = 0.007. However, there was a significant effect for Narrative Complex Language (Group (F(1, 46) = 9.696, *p* = .003, ŋ^2^ = 0.174)) as the TD participants produced more complex language than the HFASD participants. The significant Group difference was maintained when controlling for PIQ (*F*(1, 45) = 6.639, *p* = .013, ŋ^2^ = 0.129). However, there was no significant effect of Gender (*F*(1, 46) = 1.258, *p* = .268, ŋ^2^ = 0.027) and no significant Group × Gender interaction: (*F*(1, 46) = 0.068, *p* = .796, ŋ2 = 0.001). TD participants produced a significantly wider range of temporal markers than the HFASD group (Group (*F*(1, 46) = 5.271, *p* = .026, ŋ^2^ = 0.103)). This effect remained significant when controlling for PIQ (*F*(1, 45) = 5.209, *p* = .027, ŋ^2^ = 0.104). However, there was no effect of Gender (*F*(1, 46) = 0.531, *p* = .470, ŋ^2^ = 0.011) and no significant Group x Gender interaction (*F*(1, 46) = 1.795, *p* = .187, ŋ^2^ = 0.038).

TD participants also used more causal markers in narrative than HFASD participants (Group (*F*(1, 46) = 6.487, *p* = .014, ŋ^2^ = 0.124)) and showed a wider range of causal markers(Group (*F*(1, 46) = 9.784, *p* = .003, ŋ^2^ = 0.175)). This effect remained significant after controlling for PIQ (frequency of causal markers) Group (*F*(1, 45) = 4.671, *p* = .036, ŋ^2^ = 0.094) and range of causal markers (Group (*F*(1, 45) = 8.336, *p* = .006, ŋ^2^ = 0.156)). No gender effects were found for number of causal markers (frequency: (*F*(1, 46) = 0.696, *p* = .408, ŋ^2^ = 0.015); Group × Gender (*F*(1, 46) = 0.005, *p* = .942, ŋ^2^ = 0.000) or for range (*F*(1, 46) = 3.009, *p* = .089, ŋ^2^ = 0.061) or Group × Gender: *F*(1, 46) = 0.564, *p* = .456, ŋ^2^ = 0.012).

Overall for above-sentence level analyses, there were few differences between diagnostic or gender groups. There was a range of subtle group differences in Expressive Narrative, including language complexity total score, range of temporal markers used and number and range of causal markers used. In each case the HFASD group scored lower than the TD group and, unlike measures of basic structural language, this group effect was maintained after controlling for PIQ. Contrary to prediction, there were no significant differences between males and females on above-sentence level tasks.

### Measures of Pragmatic and Semantic Skills

Pragmatic language data is reported in Table 5, showing mean and standard deviations for the Figurative Language task (meaning and intent), Local Coherence Inference task (number correct and time taken) and Coherence in Narrative scores.

**Table 5:** Descriptive statistics for pragmatic language assessments for HFASD and TD groups by gender

	HFASD (n = 26) Mean (SD)	TD (n = 26) Mean (SD)	Gender overall Mean (SD)	Significance on measure		
	Raw score	Raw score		Group	Gender	Interaction
Figurative language—meaning (max score 21)				*p* < .001	p = .003	*p* = .662
Female	16.46 (2.33)	19.38 (0.87)	17.92 (2.28)			
Male	14.00 (3.42)	17.54 (2.76)	15.77 (3.54)			
Group overall	15.23 (3.13)	18.46 (2.21)	16.85 (3.14)			
Figurative language—intent (max score 20)				*p* < .001	*p* = .003	*p* = .758
Female	7.31 (4.75)	13.69 (3.28)	10.50 (5.16)			
Male	3.46 (3.10)	10.54 (4.67)	7.00 (5.30)			
Group overall	5.38 (4.39)	12.12 (4.27)	8.75 (5.47)			
Local coherence inference—total correct (max score 18)				*p* = .001	*p* = .039	*p* = .184
Female	14.62 (2.63)	16.08 (1.19)	15.35 (2.13)			
Male	12.69 (2.32)	15.54 (1.94)	14.12 (2.55)			
Group overall	13.65 (2.62)	15.81 (1.60)	14.73 (2.41)			
Local coherence inference—time (s)				*p* = .001	*p* = .470	*p* = .377
Female	160.16 (75.56)	91.19 (49.80)	125.69 (71.83)			
Male	204.21 (167.82)	86.76 (46.40)	145.48 (134.68)			
Group overall	182.18 (129.44)	88.98 (47.21)	135.58 (107.33)			
Coherence in narrative (max score 7)				*p* ≤ .001	*p* = .215	*p* = .446
Female	5.31 (0.75)	6.42 (0.90)	5.84 (0.99)			
Male	4.62 (1.85)	6.25 (0.97)	5.40 (1.68)			
Group overall	4.96 (1.43)	6.33 (0.92)	5.62 (1.38)			

Findings for measures of Figurative Language (meaning) and Figurative Language (intent) showed a similar pattern with the TD group scoring higher than the HFASD group. There was a small but significant main effect of Group for both indices (meaning: *F*(1, 48) = 21.317, *p* < .001, ŋ^2^ = 0.308 and intent: *F*(1, 48) = 36.43, *p* < .001, ŋ^2^ = 0.431). For both meaning and intent measures, there was also a significant main effect of Gender with females scoring higher than males (meaning *F*(1, 48) = 9.474, p = .003, ŋ^2^ = 0.165 and intent *F*(1, 48) = 9.851, *p* = .003, ŋ^2^ = 0.170). These effects remained significant when controlling for PIQ (meaning: Group *F*(1, 47) = 15.634, *p* < .001, ŋ^2^ = 0.250; Gender: *F*(1, 47) = 9.319, p = .004, ŋ^2^ = 0.165 and intent: Group *F*(1, 47) = 26.965, *p* < .001, ŋ^2^ = 0.365; Gender *F*(1, 47) = 10.655, *p* = .002, ŋ^2^ = 0.185). However neither group by gender interactions were significant (meaning: *F*(1, 47) = 0.193, *p* = .662, ŋ^2^ = 0.004 and intent: *F*(1, 47) = 0.096, *p* = .758, ŋ^2^ = 0.002).

On measures of coherence, a similar pattern of group and gender differences was found. For Local Coherence Inference(total correct), there was a small but significant main effect of Group (*F* (1, 48) = 13.785, *p* = .001, ŋ^2^ = 0.223) as the TD group scored higher (Mean = 15.81) than the HFASD group (Mean = 13.65).There was also a small significant main effect of Gender (*F*(1, 48) = 4.501, *p* = .039, ŋ^2^ = 0.086) with females scoring higher (Mean = 15.35) than males (Mean = 14.12). These effects remained significant when controlling for PIQ (Group: *F*(1, 47) = 7.405, *p* = .009, ŋ^2^ = 0.136; Gender: *F*(1, 47) = 5.202, *p* = .027, ŋ^2^ = 0.100). The Group × Gender interaction was not significant (*F*(1, 48) = 1.424, *p* = .239, ŋ^2^ = 0.029). Local coherence inference—time: there was a small but significant main effect of Group (*F*(1, 48) = 11.737, *p* = .001, ŋ^2^ = 0.196), as the TD participants responded faster (Mean = 88.98) than the HFASD participants (Mean = 182.18).This remained significant when controlling for PIQ (Group: *F*(1, 47) = 8.460, *p* = .006, ŋ^2^ = 0.153). There was no significant main effect of Gender (*F*(1, 48) = 0.530, *p* = .470, ŋ^2^ = 0.011) and no significant Group x Gender interaction (*F*(1, 47) = 0.794, *p* = .377, ŋ^2^ = 0.016). Coherence in narrative: There was a significant main effect of Group (*F*(1, 46) = 16.121, *p* = < 0.001, ŋ^2^ = 0.260) as the TD group (Mean = 6.33) outperformed the HFASD group (Mean = 4.96). This Group difference was maintained after controlling for PIQ (Group: *F*(1, 45) = 12.997, *p* = .001, ŋ^2^ = 0.224). There was no significant effect of Gender: (*F*(1, 48) = 0.158, *p* = .215, ŋ^2^ = 0.033) and no significant Group × Gender interaction: (*F*(1, 48) = 0.592, *p* = .446, ŋ^2^ = 0.013).

Table 6 shows raw score means and standard deviations on the CELF-4 Word Association subtest. There was a small but significant main effect for CELF-4 Word Associations of Group (*F*(1, 48) = 10.974, *p* = .002, ŋ^2^ = 0.186) as TD participants generated more category words (Mean = 54.15) than the HFASD group (Mean = 43.96). There was also a significant main effect of Gender (*F*(1, 48) = 5.465, *p* = .024, ŋ^2^ = 0.102) as females generated more category words (Mean = 52.65) than males (Mean = 45.46). These effects remained significant when controlling for PIQ (Group: *F*(1, 47) = 6.442, *p* = .015, ŋ^2^ = 0.121; Gender *F*(1, 47) = 5.494, *p* = .023, ŋ^2^ = 0.105). The Group × Gender interaction was not significant (*F*(1, 48) = 0.544, *p* = .464, ŋ^2^ = 0.011).

**Table 6:** Descriptive statistics for CELF-4 Word Association subtest for HFASD and TD groups by gender

	HFASD (n = 26) Mean (SD)	TD (n = 26) Mean (SD)	Gender overall Mean (SD)	Significance on measure		
	Raw score	Raw score		Group	Gender	Interaction
CELF-4 Word Associations subtest (max score 21)				*p* = .002	*p* = .024	*p* = .023
Female	48.69 (13.44)	56.62 (8.87)	52.65 (11.87)			
Male	39.23 (9.52)	51.69 (11.93)	45.45 (12.34)			
Group overall	43.96 (12.39)	54.15 (10.60)	49.06 (12.52)			

For the pragmatic and semantic tasks, our findings overall were of group and gender differences on understanding meaning and intent on the Figurative Language Task and on Local Coherence Inference task (total correct score) and the CELF-4 Word Associations subtest. The pattern was consistent with females outperforming males and TDs outperforming HFASDs.

### Measures of Language of Emotion

Table 7 shows descriptive data for language of emotion tasks including Receptive Emotion Vocabulary (number chosen: correct and incorrect—close and distant), Spontaneous Emotion Vocabulary in Narrative (total and range) and the Word Associations task—language of emotions.

**Table 7:** Descriptive statistics for language of emotion tasks for HFASD and TD groups by gender

	HFASD (n = 26) Mean (SD)	TD (n = 26) Mean (SD)	Gender overall Mean (SD)	Significance on measures		
	Raw score	Raw score		Group	Gender	Interaction
Spontaneous emotion vocabulary in narrative—number (max score 15)				*p* = .028	*p* = .191	*p* = .488
Female	6.08 (4.46)	7.75 (3.14)	6.88 (3.90)			
Male	3.92 (3.04)	7.08 (4.17)	5.44 (3.90)			
Group overall	5.00 (3.90)	7.42 (3.62)	6.16 (3.93)			
Spontaneous emotion vocabulary in narrative—range (max score 11)				*p* = .210	*p* = .078	*p* = .469
Female	4.54 (2.90)	4.92 (1.83)	4.72 (2.41)			
Male	2.77 (2.39)	4.17 (2.59)	3.44 (2.53)			
Group overall	3.65 (2.76)	4.54 (2.23)	4.08 (2.53)			
Word association—language of emotion (max score 18)				*p* = .930	*p* = .005	*p* = .659
Female	11.00 (2.97)	10.54 (4.01)	10.77 (3.47)			
Male	8.08 (1.94)	8.38 (3.20)	8.23 (2.60)			
Group overall	9.54 (2.87)	9.46 (3.73)	9.50 (3.29)			
Receptive emotion vocabulary (REV)—number correct (max score 36)				*p* = .044	*p* = .067	*p* = .922
Female	27.15 (4.16)	29.69 (4.01)	28.42 (4.21)			
Male	25. 08 (4.15)	27.38 (4.56)	26.23 (4.43)			
Group overall	26.12 (4.21)	28.54 (4.37)	27.33 (4.42)			
REV-incorrect close distracters (max score 13)				*p* = .887	*p* = .887	*p* = .540
Female	7.69 (3.15)	7.08 (3.20)	7.38 (3.13)			
Male	7.31 (2.36)	7.69 (2.90)	7.50 (2.60)			
Group overall	7.50 (2.73)	7.38 (3.01)	7.44 (2.85)			
REV-incorrect distant distracters (max score 13)				*p* = .026	*p* = .009	*p* = .781
Female	3.62 (1.85)	2.23 (1.64)	2.92 (1.85)			
Male	5.69 (3.35)	3.92 (2.69)	4.81 (3.12)			
Group overall	4.65 (2.86)	3.08 (2.35)	3.87 (2.71)			

There was a small significant effect on Receptive Emotional Vocabulary task (items correct) of Group (*F*(1, 48) = 4.278, *p* = .044, ŋ^2^ = 0.082) as the TD participants answered more questions correctly (mean = 28.54) than the HFASD participants (mean = 26.12). This was no longer significant after controlling for PIQ (*F*(1, 47) = 2.342, *p* = .133, ŋ^2^ = 0.047).There were no significant between differences: for Gender: (*F*(1, 48) = 3.502, *p* = .067, ŋ^2^ = 0.068); Group × Gender: (*F*(1, 48) = 0.010, *p* = .922, ŋ^2^ = 0.000); nor on Receptive Emotional Vocabulary task (items incorrect but close distracters) (Group: *F*(1, 48) = 0.020, *p* = .887, ŋ^2^ = 0.000); Gender: (*F*(1, 48) = 0.020, *p* = .887, ŋ^2^ = 0.000); Group × Gender: (*F*(1, 48) = 0.381, *p* = .540, ŋ^2^ = 0.008). For the measure Receptive Emotional Vocabulary task (items incorrect and distant distracters), there was a small but significant main effect of Gender (*F*(1, 48) = 7.511, *p* = .009, ŋ^2^ = 0.135). Females made fewer distant incorrect choices (Mean = 2.92) than males (Mean = 4.81). The significance was retained when PIQ was included as a covariant (*F*(1, 47) = 7.297, *p* = .010, ŋ^2^ = 0.134). There was also a small and significant main effect of Group (*F*(1, 48) = 5.259, *p* = .026, ŋ^2^ = 0.099) as the TD participants made less distant distracter errors (Mean = 3.08) than the HFASD participants (Mean = 4.65).However, this was no longer significant after controlling for PIQ (*F*(1, 47) = 3.330, *p* = .074, ŋ^2^ = 0.066). There were no significant effects Group × Gender interaction (*F*(1, 48) = 0.078, *p* = .781, ŋ^2^ = 0.002).

TD participants used more emotion words than HFASD participants on the Spontaneous Emotion Vocabulary in Narrative task (total) (Group (*F*(1, 46) = 5.166, *p* = .028, ŋ^2^ = 0.101) and this remained significant when controlling for PIQ (*F*(1, 45) = 7.147, *p* = .010, ŋ^2^ = 0.137). There was no significant effect of Gender: (*F*(1, 46) = 1.759, *p* = .191, ŋ^2^ = 0.037); and no significant Group x Gender interaction: *F*(1, 46) = 0.489, *p* = .488, ŋ^2^ = 0.011). However on the Spontaneous Emotion Vocabulary in Narrative task (range), there were no significant effects on this measure (Group: *F*(1, 46) = 1.615, *p* = .210, ŋ^2^ = 0.034); Gender: (*F*(1, 46) = 3.252, *p* = .078, ŋ^2^ = 0.066); Group × Gender: (*F*(1, 46) = 0.532, *p* = .469, ŋ^2^ = 0.011).

There was a small but significant main effect of Gender (*F*(1,48) = 8.606, *p* = .005, ŋ^2^ = 0.152) on the Word Association: language of emotions task. Females generated more correct emotion words (Mean = 10.77) than males (Mean = 8.23). This was maintained after controlling for PIQ (*F*(1,47) = 8.344, *p* = .006, ŋ^2^ = 0.151). But there was no significant main effect of Group (*F*(1, 48) = 0.008, *p* = .930, ŋ^2^ = 0.000) and no significant Group × Gender interaction: (*F*(1, 48) = 0.198, *p* = .659, ŋ^2^ = 0.004).

In general, our emotion vocabulary battery showed a mixed set of findings. Testing Receptive Emotion Vocabulary task revealed that there were no between diagnostic group differences. However, males tended to choose more distant distracter items than females. TD children spontaneously generated more emotion words than HFASD children, but there was no difference in the range of words used. There was an effect of gender on the number of words generated in the Word Association task (language of emotion), with females generating more words than males.

### Post-hoc Power Analysis

Where non-significant results occurred (group and/or gender), contrary to our predictions, a set of post-hoc power analyses was conducted to estimate statistical power, using GPower (Faul and Erdfelder 1992; Erdfelder et al. 1996). Sample sizes for specific tasks or subtests, α = 05, and effect sizes (ŋ^2^) from individual measures were used to estimate power (1 − β): shown in Table 8. For some measures, only gender or only group differences were predicted, so analysis focused on the relevant comparison. In all cases, the post-hoc power analysis indicated a lack of statistical power. This is likely due to small effect sizes which exist throughout this data subset. It is possible that future research which included a large participant group may find between group or gender differences.

**Table 8:** Post-hoc power values for selected pragmatic and emotion word tasks

Task or subtest	Estimated power and effect size	
	Between groups 1 – β (ŋ^2^)	Between gender 1 – β (ŋ^2^)
CELF understanding spoken paragraphs	0.059 (0.022)	0.068 (0.044)
Expressive language sample in narrative (story length)	0.079 (0.065)	0.070(0.047)
Expressive language sample in narrative (complexity)		0.061(0.027)
Coherence in narrative task		0.063(0.033)
Receptive emotional vocabulary task (items correct)	0.088(0.082)	0.080 (0.068)
Receptive emotional vocabulary task (distant distracters)	0.098(0.099)	
Spontaneous emotion vocabulary in narrative task (total)		0.065(0.037)
Spontaneous emotion vocabulary in narrative task (range)	0.064(0.034)	0.079(0.066)
Word associations (language of emotion) task	0.050(< 0.001)	

## Discussion

Findings from a large set of established and novel language and pragmatics tasks indicated some interesting features of communication for females with HFASD. The main significant findings are that, for receptive pragmatic, semantic and language of emotion measures conducted in this study, FwHFASD show significant difficulties compared to FwTD, but are less impaired than MwHFASD.

We found significant effects for gender and diagnostic group on Figurative Language (meaning), Figurative Language (intent) and Local Coherence Inference (total correct). To our knowledge this is the first gender comparison on these types of tasks. TDs outperformed HFASDs, as expected from the findings of the task creators (MacKay and Shaw 2004, Joliffe and Baron-Cohen 1999). We also found subtle gender differences on the CELF-4 Word Associations subtest. Females with HFASD in our study recalled more words within category on this task compared to males with HFASD. Overall TDs performed better than HFASD. These outcomes are in line with those from previous work by Goddard et al. (2014) and perhaps confirms the hypothesis that children/young people with TD have more extensive semantic knowledge, presumably founded on more robust word learning in the earlier years (Bowler et al. 2008). Anecdotally, we noted a difference in strategies for naming category words between the males and females with HFASD, with males seemingly more likely to have intrusions from special interests (e.g., entomologist as a type of occupation) and listing (12 types of shark in the category animals). Taken together these findings imply that FwHFASD show significant difficulties with some aspects of complex pragmatic and semantic functioning compared to FwTD, but are less impaired than MwHFASD.

Individuals with HFASD produced less complex and less coherent narration than TDs. However, it is surprising that no difference was found between genders on this measure. The small participant numbers used in this study would lead to lower statistical power and it is possible that gender differences may be identified in a larger population sample. Isolated pragmatic tasks generally may artificially alter the social context of the task and thereby limit its use in evidencing functional communication difficulties (Adams 2002). A comparative analysis between genders using measures of pragmatic features in discourse would be a useful additional investigation. Also a comparison of outcomes on isolated receptive tasks (reported here) and functional pragmatic ability in discourse could provide important insight into the validity of using such tasks to identify functional deficit.

Overall we found consistent results across all receptive pragmatic and semantic measures, with females performing better than males and TDs performing better than HFASDs. Numeric scores on pragmatic and semantic tasks showed a trend for females with TD to perform best, followed by males with TD, then females with HFASD, and finally males with HFASD tending to perform worse on all measures.

The above-sentence level comprehension tasks in our study did not find differences between groups or gender. In terms of expressive language at above-sentence level we found some group, but not gender differences across a range of tasks. Using a composite tally of temporal and causal markers in narrative (following Petersen et al. 2008), and controlling for verbal IQ, we found that HFASD groups had a lower language complexity score than TDs, similar to findings of Eigsti et al. (2007), Kelley et al. (2006) and Scarborough et al. (1991). Our study found no difference between gender or diagnostic group in terms of story length, which is in line with previous work (Diehl et al. 2006). It is also possible that gender differences within higher level structural language may be evident in a study with a larger sample size.

Our study provides evidence of significant differences in all three measures of emotion vocabulary: the Receptive Emotional Vocabulary task, the Word Association: language of emotions task and the spontaneous generation of emotional vocabulary in narrative. As expected, TD performed better than HFASD across measures. Overall findings suggest that FwHFASD are more likely than MwHFASD to accurately understand and use emotion vocabulary, but that they will be impaired by comparison to their typically developing female peers. Generally, the difference between females and males with HFASD in our study is similar to the difference demonstrated between female/males with TD, mediated by diagnosis. However, on two measures FwHFASD performed on a par with FwTD showing a similar skill gap to males either with or without autism. This may then represent a naturally occurring advantage to females mediated by HFASD diagnoses.

We found significant differences according to diagnostic group (TD > HFASD), but not gender, on the total number of emotion terms in spontaneous narrative, similar to previous research (Perlman-Avnion and Eviatar 2002; Sillar et al. 2014). However, a female advantage in this task would be predicted from TD population studies (Thompson and Moore 2000; Newman et al. 2008). This negative finding and the lack of diagnostic group difference in the range of emotional vocabulary used might indicate our task was insufficiently sensitive to detect differences of this type. By asking a child to re-tell a story from a book they were primed with the same picture references. This may support individuals with lower natural inclination to talk about emotions. A tally of emotion words in free conversation may therefore provide more elucidating results.

In contrast there was a clear female advantage replicated across diagnostic groups on the task of generation of words within the semantic category of emotions. Again, it is surprising that HFASDs did not show limitations compared to TDs on this task. Gaffery et al. (2007) found that HFASDs were relatively impaired in identifying category membership, most notably in the category of feelings. Similarly, using a novel measure of Receptive Emotional Vocabulary, there was no difference between HFASD and TD group findings, which would be expected from previous research (Hobson and Lee 1989). The total number of items (both correct and incorrect) was the same across genders. However, males (TD and HFASD) were more likely to choose grossly incorrect picture representations for emotional vocabulary than either female diagnostic group. This represents a subtle but important gender difference, suggesting that females may be better able to make closer estimations of word meaning in later emerging vocabulary of emotion. It is worth remembering that the Word Association—language of emotion and Receptive Emotional Vocabulary tasks were novel to this study, and so have not been validated on broader samples. Nonetheless, it is surprising no difference was found on either measure between HFASD and TD groups, as this is thought to be a common area of difficulty for the diagnosed group (Frith 1991). It is possible that the high cognitive ability of our participants may mediate performance in this area. It is also possible that other factors of facial and visual recognition, processing, understanding and production (Lartseva et al. 2015) may be more impactful than vocabulary in emotional intelligence. Post hoc power analysis also showed low statistical power across the range of language of emotion tasks, therefore increasing the possibility of incurring a type II error. It is important for further investigation to be undertaken with larger participant samples in this area, in order to clarify ambiguities in the current findings.

That we found no difference in expressive or receptive language tasks at basic structural language level between gender or diagnostic group is commensurate with previous research (Asberg 2010, Howlin 2003; Kjelgaard and Tager-Flusberg 2001; Kelley et al. 2006). Before the existence/non-existence of language difficulties can be confirmed within this group of older children with HFASD and TD we note that tests should be selected that can demonstrate the appropriate breadth of difficulty. For example, scores on TROG reached ceiling, precluding variance required for comparisons. The novel structural level task, Sensitivity to Grammatical Errors, with which Eigsti and Bennetto (2009) demonstrated a TD > HFASD advantage, did not show group or gender differences in the current study. It is possible that our amendments to the original test material (including reduced number of test items) may account for our findings.

### Creating a Profile of Language and Communication Difficulties for Female HFASD

Importantly, our study indicates the existence of a specific profile of performance in the core autism diagnostic domain of language and communication, and supports related research which has identified differences in isolated communication skills (Holtmann et al. 2007; Park et al. 2012; Hiller et al. 2014). It is also in line with findings from other core diagnostic domains: social interaction (Head et al. 2014) and restricted, stereotyped, repetitive behaviours (Mandy et al. 2012). Our profile tentatively provides a summary of findings, with a focus on the presentation of females with HFASD. However, it is important to note that little evidence from the wider literature exists on the specific tasks used in this study and all strengths and weaknesses identified here would benefit from further robust investigation in larger scale studies.

1. Females with HFASD have similar expressive and receptive vocabulary and sentence level language to males with HFASD and TD controls.
2. Female with HFASD show subtle deficits in higher level structural language tasks (expressive language in narration) when compared to TD controls (female and male). However, male HFASD have a similar profile in this language domain.
3. Females with HFASD perform better than males with HFASD in a range of pragmatic language and semantic tasks. However, they perform worse than females with TD.
4. Females with HFASD perform similarly to females with TD on some language of emotion measures (receptive and semantic category naming) and better than males with TD or males with HFASD. These may represent relatively spared skills compared to gender norms.
5. Females with HFASD appear to perform worse than females with TD when using spontaneous vocabulary of emotion in narration. In this respect they perform similarly to males with HFASD.

### Clinical Implications and Conclusions

A distinct profile of language and communication skills for females with HFASD has significant implications within clinical practice. Comparably preserved skills in pragmatics, semantics and language of emotion may mean subtle difficulties are insufficient to meet criteria using current diagnostic tools (Gould and Ashton-Smith 2011).If females with HFASD perform better than males with HFASD they may appear more socially ‘savvy’ to observers in clinical services and diagnostic teams, and consequently may not meet current diagnostic criteria for difficulties with social communication. Later age diagnosis will result in poorer access to services, increased vulnerability to risks and reduced well-being (Bargiela et al. 2016). However, subtle differences in pragmatic ability will impact on an individual’s quality of social interactions and their capacity to interpret the intentions of others (Dennis et al. 2001). Our results indicate that in comparison with females with TD, girls with HFASD may be unable to perform on a par with peers within social situations, affecting their ability to make and maintain friendships. Appropriate and timely access to therapeutic services targeting subtle communication difficulties could help mediate these difficulties.

In conclusion, the current study contributes to the existing literature by indicating that females with HFASD differ in their presentation of autism symptomology from male HFASD. This may confound their accurate diagnosis and contribute to poorer diagnostic rates. It also raises the important possibility that the same females with HFASD are experiencing a social disadvantage when compared to females with TD. Without a timely diagnosis, access to suitable therapeutic services will be unmet. Lack of appropriate diagnosis and therapeutic support, with potential social impact, could contribute to the recognised increase in mental health difficulties experienced within the females with autism group.

Potential clinical implications of above-sentence level difficulties found in this study will impact on social functioning and presentation for both females and males with HFASD. For example, one implication of using fewer causal markers at the above-sentence level might be impoverished sequencing of related information in narrative and discourse. Poor sequencing of narrative information will directly impact on conveying a point of view (Sillar et al. 2014), which will in turn impact on the individual’s ability to self-advocate. In addition, a narrower range of vocabulary choices in connected speech will also mark the speaker as less skilled than her peers and subtly less linguistically mature. These difficulties are likely to impact on integration into female TD friendship groups (Dean et al. 2013). Bespoke support could be beneficial in mediating that need, and potentially prevent the emotional impact of peer rejection.

Wider implications for clinical practice revolve around the use of language measures. While basic structural measures may be a suitable tool to either rule in or out severe language impairment, this study found them to be insufficient in evidencing the above-sentence language difficulties associated with HFASD for either gender. Children who scored comparably at word and sentence level showed significant functional difficulties in higher structural language tasks as well as pragmatic and semantic measures. Further research is required to better understand this discrepancy in HFASD profiles. Clinically, even fairly crude measures of language complexity (counting causal and temporal markers) provided better evidence of subtle difference between HFASD and TDs at this level and may be more useful to evidence functional difficulties.

### Limitations and Future Research

Caution in interpretation is entailed due to the small samples in the current study, raising the risk of type II errors. Effect sizes were also small throughout, which may be indicative of the nature of language differences between genders. However, null results are difficult to interpret. A lack of statistical significance in this study should not be interpreted as a lack of potential difference which may be evidenced in a larger group sample. Although the pattern of findings (especially for pragmatics and semantics tasks) showed consistencies across multiple measures, a larger scale project should be conducted to establish the validity of these trends. Secondly, many of the items used in testing were experimental, and two were novel. Additional normative data should be collected from TD and clinical populations in order to ascertain the effectiveness of these tasks in measuring target skills and replicate the current findings. Finally, participants were carefully selected to represent females who were least likely to receive a diagnosis (in particular those with a higher IQ and diagnosed at a later age; middle childhood). It was the experience of the researchers that participants matched descriptions of the *missed diagnosis group*. However, our participants *did* have a diagnosis and so may still present with a male-type profile. A next step in research may be to apply our research findings to a wider group; for example, girls meeting criteria from an undiagnosed at-risk sample group (siblings of children with autism) or whole population cohorts.

We argue that this study provides novel preliminary data in the field of HFASD and communication and points to important areas of difference across diagnostic groups and gender, signposting fruitful avenues for future research and intervention. It contributes to the existing body of work currently informing diagnostic assessment and intervention for language and communication difficulties for children with HFASD. It also introduces useful themes for further investigation regarding the female HFASD profile and how this differs from male HFASD and gender normative data. It indicates the need for larger scale studies in this area.

## Electronic supplementary material

Below is the link to the electronic supplementary material.

Supplementary material 1 (DOCX 25 KB)

## Footnotes

1 for brevity in results, females/males with HFASD/TD will be referred to as FwHFASD/MwHFASD/FwTD/MwTD.
