# Supplementary material for: Speaking the Same Language? A Preliminary Investigation, Comparing the Language and Communication Skills of Females and Males with High-Functioning Autism
Source: J Autism Dev Disord. 2019 Mar 4;50(5):1639–56. doi: 10.1007/s10803-019-03920-6 (PMC7211208; doi:10.1007/s10803-019-03920-6)
Supplement: Supplementary file 1 — Supplementary material 1 (DOCX 25 KB) [file 10803_2019_3920_MOESM1_ESM.docx]

Appendix

*Appendix 1: Adaptations to sensitivity to grammatical errors task*

Original material by Eigsti & Bennetto (2009) was adapted in the following ways:

- Number of test items were reduced from 140 to 72 (38 grammatical, 38 matched ungrammatical) to manage test timings
- Items with grammatical violations relating to word order, particle movement , yes no questions and wh questions were removed due to lack of difference between HFASD and TDs in original test material
- All remaining violation categories were represented by two test items making 18 in total

| **Grammatical rule violation type & no.** | **Sample grammatical sentence** |
| --- | --- |
| Past tense – irregular (n: 2) | Last week the baby throwed a cat into the bath tub |
| Past tense – irregular aux (n: 2) | Over the winter Martha had feeled sick |
| Plural –s not irregular (n: 2) | A shoe salesman sees many foots throughout the day |
| Present prog omission (n: 2) | Janet is wear the dress I gave her |
| Determiner omission (n: 2) | Girl played with her friend outside |
| Erroneous possessive pronoun (n: 2) | Carol is cooking dinner for hers family |
| Particle omission (n: 2) | They stood the line very patiently |
| Unmarked particle for aux ‘be’ (n: 2) | Fred will be get a raise next month |
| Past tense omitted (n: 2) | Last night the old lady die in her sleep |
| Verb temporal marker (n: 2) | Right now Judy shopped for a new party dress |
| Plural omission (n: 2) | Many house were destroyed by the flood last week |
| 3^rd^ person singular omission (n: 2) | Everyday Suzy talk with her mum on the phone |
| Determiner insertion (n: 2) | Larry went the home after the party |
| Mismatch case and pronoun (n: 2) | The girl is making some cookies for we |
| Mismatch agreement of pronouns (n: 2) | The girl cut himself on a piece of glass |
| Unmarked particle for aux ‘have’ (n: 2) | The baby bird has fall from the oak tree |
| Under extension of tense marking (n: 2) | Leonard should has written a letter to his mother |
| Inappropriate wh- question (n: 2) | Why did she put the book? |

Example of paired grammatical and ungrammatical equivalent:

“Every day Lucy talk with her mum on the phone” (third person singular omission)

“Every day Jack walks with his friend in the park” (grammatical equivalent)

*Appendix 2: Pragmatics: Figurative language task*

Original material by (MacKay & Shaw, 2004) was adapted in the following ways:

- Number of test items for figurative the original list of language forms( irony, hyperbole, metonymy, indirect comment, rhetorical question, understatement) were reduced from 10 repetitions of each item, to 3 repetitions of each item. Resulting in 18 test items
- Three examples of metaphor were also added yielding 21 test items.
- Metaphor examples were matched to original material in terms of story length and word appropriacy for age group
- Materials were presented on a Power Point presentation rather than pieces of paper
- Images were taken from the internet and identified as successfully representing the true meaning of the figurative language sample
- All adaptations were peer reviewed to ascertain appropriacy for the participant population

| **Figurative language type & no.** | **Sample sentence** |
| --- | --- |
| Hyperbole | You have millions of CDs (lots of) |
| Indirect Requests | It’s very noisy out there (be quiet) |
| Metonymy | Quieten down sandpit (children in the sandpit) |
| Irony  Rhetorical Questions | Great singing mum (dreadful singing)  Are you trying to get yourself killed? |
| Understatement | Just a few things (lots of shopping) |
| Metaphor | My teacher is a dragon (horrible) |

*Appendix 3: Pragmatics: Local Coherence Inference task*

Original material by Joliffe and Baron-Cohen (1999) was adapted in the following ways:

- 18 test items were derived from the six presented in the original study design
- New test items were matched for sentence length
- All vocabulary choices, language complexity and topics were tested for age appropriacy with a TD middle childhood pilot study.
- Test items were found to be appropriate for the age group and there were no floor or ceiling effects in results.
- Minor adaptations were made following feedback.

*Appendix 4: Language of emotions: Receptive Emotional Vocabulary (REV)*

In response to the lack of established testing materials suitable to measure receptive vocabulary of emotion a tool conceptually similar to the BPVS (Dunn et al, 1997) was developed. Participants would be shown four pictures of faces, each representing an emotional state. They would then be presented with a spoken target word and asked to identify the matching picture. Images for the assessment were sourced from Baron-Cohen’s Mind Reading Emotions Library (2004).

Target words for the test stimuli were initially identified using Baron-Cohen et al (2010) catalogue of 366 emotion and mental state words, which also provides data on age of word emergence. Words were considered established when they could be correctly used by 70%+ of the children surveyed. The word list was filtered to exclude words that required an action, situation or interactive component which could not easily be represented in a picture of a face. The list was further refined to eliminate words that were based on the opinions of a third person i.e. “liked”. Words with a secondary literal meaning i.e. “warm” or “bitter” were also excluded to avoid additional difficulties the children with ASD may experience due to non-literal meaning. The remaining list was distilled to 33 items with proportional numbers of words developed across age brackets (4-6, 7-8, 9-10, 11-12, 13-14, 15-16) and valance (positive, negative, neutral).

Each slide presents one target word, one close distracter, one distant distracter and one randomly assigned but not close distracter. Close distracters were selected from the same developmental bracket and same valance group (i.e. positive, negative or neutral emotion) but not the same emotional category (i.e. grumpy and angry both fall under the same emotion category of angry). Distant distracters were also chosen from the same developmental age bracket but not the same valance or emotion group. Valance and emotion categories were established by Baron-Cohen et al (2010). The final materials were evaluated by naive experimenters to assure correct decisions had been made about target words, picture choices and closeness of distracters. A final list of target words and close distracters is tabulated below:

| **No.** | **Target word** | **Close distracter** | **No.** | **Target word** | **Close distracter** |
| --- | --- | --- | --- | --- | --- |
| Practice | Grumpy | - | 17 | Startled | Anxious |
| 1 | Happy | Friendly | 18 | Frustrated | Unfriendly |
| 2 | Sad | Worried | 19 | Affectionate | Friendly |
| 3 | Afraid | Disgusted | 20 | Enthusiastic | Happy |
| 4 | Angry | Worried | **21** | Stubborn | Baffled |
| 5 | Surprised | Interested | **22** | Distant | Embarrassed |
| 6 | Worried | Sad | **23** | Determined | Aggressive |
| 7 | Disgusted | Angry | **24** | Adoring | Kind |
| 8 | Confused | Worried | **25** | Baffled | Unsure |
| 9 | Calm | Friendly | **26** | Humiliated | Anxious |
| 10 | Proud | Overjoyed | **27** | Contemptuous | Unfriendly |
| 11 | Embarrassed | Afraid | **28** | Choosing | Guilty |
| 12 | Thoughtful | Confused | **29** | Intimidated | Worried |
| 13 | Overjoyed | Surprised | **30** | Mystified | Confused |
| 14 | Threatened | Surprised | **31** | Empathic | Enthusiastic |
| 15 | Guilty | Unfriendly | **32** | Indifferent | Stubborn |
| 16 | Amused | Friendly | **33** | Stern | Confused |

A small pilot study was conducted to investigate face validity and utility of this measure before inclusion in this research. 54 typically developing children were recruited covering a range of age bands: 8-8:11 (n:13), 9-9.11 (n:7), 10-10:11 (n:14), 11-11:11 (n:5), 12-12:11 (n:10), 13-13:11 (n:5). Results indicate there were no significant difficulties administering the material or engaging the children in the activity. In addition, the stimuli showed a range of scores and no notable floor or ceiling effects for any of the age groups investigated. A descriptive analysis of data indicates an age effect, with accuracy improving consecutively (i.e. proportional diminishing of close and distant distracters).

*Appendix 5:* Language of emotions: *Semantic word association task*

This novel measure was based on the CELF-4 semantic word association task (Semel et al, 2006). Participants are asked to generate as many words as possible from the category emotions within 60 seconds. Responses were recorded for analysis and transcribed. The child is told:

“Name different feelings or emotions that people might have. Name as many as you can in 1 minute. For example you could say *happy* or *embarrassed*. Now you name some more. Start now”

Results were scored in line with CELF-4: responses should be real, a correct representation of the category heading and not repeated. Judgement on whether a word belonged to the category emotions was based initially on Baron-Cohen et al (2010) and where necessary the Oxford English dictionary (Oxford University Press, 2010). Correct items are tallied to provide a raw score. A small pilot study with 54 typically developing children found the material to be appropriate for measuring word association for language of emotion across a range of age groups (8.00 to 13.11 years) with no floor or ceiling effects.
